# Health self-perception is associated with life-styles and comorbidities and its effect on mortality is confounded by age. A population based study

**DOI:** 10.3389/fmed.2022.1015195

**Published:** 2022-11-23

**Authors:** Oscar Rañó-Santamaría, Carmen Fernandez-Merino, Ana Isabel Castaño-Carou, Óscar Lado-Baleato, María José Fernández-Domínguez, Juan Jose Sanchez-Castro, Francisco Gude

**Affiliations:** ^1^Faculty of Medicine, Santiago de Compostela, Spain; ^2^A Estrada Primary Care Center, A Estrada, Spain; ^3^Bertamiráns Primary Care Center, Ames, Spain; ^4^Research Methods Group (RESMET), Health Research Institute of Santiago de Compostela (IDIS), Santiago de Compostela, Spain; ^5^ISCIII Support Platforms for Clinical Research, Health Research Institute of Santiago de Compostela (IDIS), Santiago de Compostela, Spain; ^6^Leiro Primary Care Center, Leiro, Spain; ^7^Health Research Institute (IDIS), Santiago de Compostela, Spain; ^8^Clinical Epidemiology Unit, University Clinic Hospital, Santiago de Compostela, Spain

**Keywords:** health self-perception (HSP), chronic diseases, mortality, lifestyles, primary care

## Abstract

**Background:**

Health self-perception (HSP) is the individual and subjective concept that a person has of their state of health. Despite its simplicity, HSP is considered a valid and relevant indicator employed in epidemiological research and in professional practice as an overall measure of health.

**Objectives:**

(1) To describe and analyze the associations between HSP and demographic variables, lifestyle and diseases prevalent in a population and (2) to investigate the relationship between HSP and mortality.

**Materials and methods:**

In a primary care setting, we conducted a longitudinal study of a random populational sample of a Galician municipality, stratified by decade of life. A total of 1,516 adults older than 18 years, recruited by the 2013–2015 AEGIS study, were followed-up for more than 5 years. During the clinical interview, data were collected on lifestyle and prevalent diseases. The HSP was grouped into 2 categories (good/poor). The statistical analysis consisted of a logistic regression, Kaplan–Meier curves and Cox regression.

**Results:**

A total of 540 (35.6%) participants reported poor HSP. At the end of the follow-up, 78 participants had died (5.1%). The participants with increased age and body mass index and chronic diseases (anxiety, depression, ischemic heart disease, diabetes, and cancer) presented a poorer subjective health. A high level of physical activity and moderate alcohol consumption were associated with better HSP. A poorer HSP was associated with increased mortality, an association that disappeared after adjusting for the rest of the covariates (HR, 0.82; 95% CI 0.50–1.33).

**Conclusion:**

(1) Health self-perception is associated with age, lifestyle, and certain prevalent diseases. (2) A poorer HSP is associated with increased mortality, but this predictive capacity disappeared after adjusting for potential confounders such as age, lifestyle, and prevalent diseases.

## Introduction

Health self-perception (HSP) is based on an individual and subjective concept that allows a person to assess their overall state of health. This measure enables a simple evaluation of the patient’s physical and emotional state. Despite its simplicity, HSP can show certain hitherto unknown conditions that could lead to increased morbidity and mortality.

Various studies have shown how individuals tend to assess their health differently depending on certain characteristics such as age, sex, education, culture, personality and even the generation to which they belong (1). HSP has significant importance to older adults, given that this group presents more morbidity and mortality, which is frequently associated with deteriorating functional capacity, resulting in an increased use of health services. These limitations can cause a depressive state in patients, which usually results in a poor perception of health (2). Socioeconomic factors, employment status and educational level have also been associated with self-rated health. Similarly, a poor health self-perception has been specifically related to a poor lifestyle, such as a lack of physical activity and obesity (3, 4).

Cohort studies have found that HSP is a significant predictor of mortality (5, 6). The relationship between HSP and all-cause mortality has been described as strong, offering better predictions than other scores designed for this purpose (7). Numerous research studies have also related poorer HSP with the presence of chronic diseases and comorbidities, as well as increased demand for healthcare services (8). Most of these cohort studies have been conducted with patients older than 60–65 years (9–11). Few studies have been conducted with representative samples of the general population. Those that have, however, found no association between HSP and mortality (12). For a better understanding of the mechanisms by which HSP is related to short and long-term mortality, we have to consider the potential confounding effects of age and comorbidities. Tamayo-Fonseca et al. (13) discussed in their study the worrying increase in negative self-perception, which along with the aging of the population can predict a change in certain objectives indicators of health (mortality, use of services, and disability). Based on a follow-up of a 4-year cohort study, the authors estimated a statistically significant effect of HSP on mortality in a Spanish population.

In this study, we studied HSP in a representative sample of the general adult population of a rural municipality with a follow-up longer than 5 years. The study objectives were (1) to determine the effect of the most common demographic variables, lifestyle, and comorbidities on HSP; (2) to estimate the effect of HSP on patient mortality; and (3) to determine its predictive value, taking into account the most common sociodemographic factors, lifestyle, and certain morbidities.

## Materials and methods

### Study design

The data source for the present study was the AEGIS study (“A Estrada Glycation and Inflammation Study”), performed in the municipality of A Estrada (Spain). AEGIS was a prospective observational study with a sample of 1516 [678 men (45%) and 838 women (55%)] individuals selected using randomized sampling stratified by age of the population of origin. The data began to be recorded in December 2012 and ended in March 2015. The participants were then followed-up until November 2020, recording the development of diseases or exitus.

Participants were selected using randomized sampling stratified by decade of life, based on health card registration, which has a coverage of greater than 95% of the population. The inclusion criteria were adults (>18 years of age), Spanish fluency or the ability to communicate and grant their informed consent. The exclusion criteria were a change in residence, having a terminal illness, not having the ability to grant informed consent, and difficulties traveling while participating in the study. All patients were scheduled for a clinical interview in A Estrada Primary Care Health Center during which blood tests, urine tests, electrocardiogram, questionnaires, and a characterization of the diagnosed diseases recorded in their medical history were performed.

Health self-perception was measured using the first question of the Short Form (SF-36) questionnaire (14) “In general, would you say your health is,” which has the following possible answers: excellent, very good, good, fair, and poor. To compare with other studies, we recoded this variable as good (excellent, very good, and good) or poor (fair and poor) HSP.

The physical activity level was assessed using the International Physical Activity Questionnaire–Short Form (15), which measures the activity performed by the patient in the past 7 days. We also recorded the tobacco (cigarettes per day) and alcohol consumption [standard drink units (SDU) per week]. In the study, we converted the collected data in grams of alcohol/week. Depending on the consumption, we classified the participants into 4 categories: 0–9 g/week, 10–139 g/week, 140–279 g/week, and ≥280 g/week.

Information about most prevalent chronic diseases (i.e., diabetes mellitus, ischemic heart disease, and cancer) was extracted from electronic health records of patients according to International Classification of Primary Care codes (ICPC-2). Additionally, this data was confirmed by a personal interview. To assess the patients’ emotional state, we used the Goldberg anxiety and depression scale in its Spanish-validated version by Montón-Franco et al. (16). We considered scores ≥2 as probable cases of depression and scores ≥4 as probable cases of anxiety.

### Statistical analysis

The qualitative variables are expressed as absolute frequencies (and percentages), while the continuous variables are expressed as means [standard deviation (SD)]. To check whether there were differences between good and poor HSP, we used the chi-squared test for qualitative variables and Student’s *t*-test or the Mann-Whitney *U* test for continuous variables.

To assess the association between HSP and the demographic variables, morbidities and lifestyle, we used multivariate logistic regression models. The models were first adjusted by age, and then all the potential predictors were introduced into the model. In order to account for the age stratified sampling, a design-based analysis was performed. The sampling procedures used in the study departed from unequal probability selection. Compensatory weights were developed to obtain estimates [prevalences and odds ratios (OR), with their corresponding 95% confidence intervals (CI)] from the original target population in the study area.

To study the association between HSP and mortality, we conducted a univariate survival analysis using the Kaplan–Meier estimator, comparing the differences between groups with the log-rank test. The study also employed a Cox proportional hazards model, adjusting the effect of each variable by age and then taking into account all demographic variables along with HSP. Based on the coefficients of the Cox regression models, we calculated the hazard ratios (HR) and their 95% CI. Compensatory weights were also considered.

The statistical analysis employed the *rms* (17) and *survival* package (18), graphic representations with *ggplot2* (19) that work within R software (20), which is freely available at https://cran.r-project.org/.

### Ethical considerations

The present study was reviewed and approved by the Clinical Research Ethics Committee from Galicia, Spain (CEIC2012-025). Written informed consent was obtained from each participant in the study, which conformed to the current Helsinki Declaration.

## Results

As shown in Table 1, the patients had a mean age of 52.6 (SD, 17.6) years, ranging in age from 18 to 88 years. The sample consisted of 55.3% women and 44.7% men. Of the 1,516 participants, 296 (19.5%) reported smoking at least 1 cigarette a day, 24.5% indicated high alcohol consumption (≥140 g/week), 39.4% reported moderate consumption (10–139 g/week) and only 36% were abstainers or mild consumers (0–9 g/week). Some 37.9% of the patients had excess weight, and 34.2% had obesity, with a mean body mass index (BMI) of 28.2 (SD, 5.1) kg/m^2^. The most prevalent diseases included in the analysis were those related to mental health: 24.5% for depression and 22% for anxiety. Diabetes mellitus was third place with 12.3%.

**TABLE 1:** Sociodemographic, lifestyle, and comorbidities for the overall sample and for good and poor self-rated health.

	AEGIS sample *n* = 1516	Good HSP *n* = 976	Poor HSP *n* = 540	*P*
Age, mean (SD)	52.6 (17.6)	48.3 (17.2)	60.4 (15.3)	< 0.001
**Sex**				
Male, n (%)	678 (44.7)	462 (47.3)	216 (40.0)	0.006
Female, n (%)	838 (55.3)	514 (52.7)	324 (60.0)	
BMI, kg/m^2^	28.2 (5.1)	27.4 (4.8)	29.8 (5.2)	< 0.001
**Physical activity**				< 0.001
Low, n (%)	596 (39.3)	339 (34.7)	257 (47.6)	
Mid, n (%)	552 (36.4)	357 (36.6)	195 (36.1)	
High, n (%)	368 (24.3)	280 (28.7)	88 (16.3)	
**Smoking status**				0.001
No, n (%)	825 (54.4)	504 (51.7)	321 (59.4)	
Ex, n (%)	395 (26.1)	256 (26.2)	139 (25.8)	
Current, n (%)	296 (19.5)	216 (22.1)	80 (14.8)	
**Alcohol**				< 0.001
0–9 g/week, n (%)	546 (36)	322 (33.0)	224 (41.5)	
10–139 g/week, n (%)	598 (39.4)	423 (43.3)	175 (32.4)	
140–279 g/week, n (%)	241 (15.9)	149 (15.2)	92 (17.0)	
>280 g/week, n (%)	131 (8.6)	82 (8.4)	49 (9.1)	
DM, n (%)	187 (12.3)	68 (6.9)	119 (22.0)	< 0.001
IHD, n (%)	65 (4.3)	21 (2.1)	44 (8.1)	< 0.001
Cancer, n (%)	71 (4.7)	26 (2.6)	45 (8.3)	< 0.001
Depression, n (%)	372 (24.5)	160 (9.2)	212 (25.5)	< 0.001
Anxiety, n (%)	334 (22.0)	168 (17.3)	166 (30.9)	< 0.001
Exitus, n (%)	78 (5.1)	39 (3.9)	39 (7.2)	0.009

BMI, body mass index; DM, diabetes mellitus; IHD, ischemic heart disease; HSP, self-rated health.

### Factors involved in poor health self-perception

Table 1 shows the characteristics of the patients with poor and good HSP. There was a predominance of patients with good HSP (976, 64.4%), while 540 (35.6%) indicated poor health perception. The women reported a poor HSP compared with 31.9% of the men (OR, 0.74; 95% CI 0.59–0.91). As age increased, the proportion of participants with a poor HSP tended to increase. The mean age was 48.3 years (17.2) for the patients with good HSP and 60.4 (15.3) years for those with poor HSP.

Table 2 shows the univariate and adjusted odds ratios for predicting poor HSP. With each year, the probability of presenting poor HSP increased by 4.4% (OR, 1.04; 95% CI 1.03–1.05). BMI was also associated with HSP. Some 47.5% of the participants with obesity reported a poor health condition, compared with 22.2% of those with a normal weight (OR, 1.09; 95 CI% 1.07–1.12). A moderate level of physical activity behaved as a protective factor for HSP (OR, 0.72; 95% CI 0.56–0.91). However, a high physical activity level was associated in the univariate analysis with a poor HSP (OR, 1.03; 95% CI 1.03–1.04). The patients who smoked stated a better HSP (OR, 0.58; 95% CI 0.43–0.77). After comparing the participants who abstained or were sporadic drinkers against the consumers, an association between abusive alcohol consumption (≥280 g/week) and HSP (OR, 0.85; 95% CI 0.58–1.27) could not be established. The weekly consumption of 10–139 g, however, appeared to be a statistically significant protective factor against poor HSP (OR, 0.59; 95% CI 0.46–0.76), with a risk reduction of 41.5%.

**TABLE 2:** Univariate and adjusted odds ratios for poor self-rated health and their 95% confidence interval.

	Univariate analysis		Adjusted for age		Multivariate model	
			
	Odds ratio	95% CI	Odds ratio	95% CI	Odds ratio	95% CI
Age	1.04*	1.04–1.05	–	–	1.03*	1.02–1.04
Male sex	0.74*	0.59–0.91	0.77*	0.61–0.96	1.01	0.75–1.36
BMI	1.10*	1.07–1.12	1.06*	1.04–1.09	1.06*	1.03–1.08
Physical activity						
Low	Ref.				Ref.	
Intermediate	0.72*	0.57–0.91	0.73*	0.56–0.94	0.83	0.64–1.10
High	0.39*	0.29–0.53	0.53*	0.39–0.72	0.67*	0.48–0.94
Smoking						
No	Ref.		Ref.		Ref.	
Ex	0.85	0.66–1.09	0.85	0.65–1.11	0.90	0.66–1.23
Current	0.56*	0.41–0.75	1.07	0.77–1.48	1.17	0.82–1.68
Alcohol						
0–9 g/week	Ref.		Ref.		Ref.	
10–139 g/week	0.58*	0.45–0.74	0.63*	0.48–0.81	0.65*	0.49–0.87
140–279	0.89	0.65–1.22	0.68*	0.49–0.96	0.77	0.53–1.12
g/week	0.89	0.59–1.33	0.73	0.47–1.11	0.84	0.51–1.36
>280 g/week						
DM	3.35*	2.53–4.45	1.99*	1.47–2.68	1.77*	1.29–2.45
IHD	3.86*	2.33–6.56	1.78*	1.05–3.09	1.80*	1.01–3.25
Cancer	3.08*	1.92–5.03	1.61	0.97–2.68	1.72*	1.01–2.96
Depression	3.43*	2.56–4.62	2.96*	2.18–4.04	2.38*	1.71–3.32
Anxiety	2.27*	1.78–2.91	2.70*	1.04–2.07	2.46*	1.85–3.28

*Indicates statistically significant odds ratios for *p* < 0.05. BMI, body mass index; DM, diabetes mellitus; IHD, ischemic heart disease; HSP, self-rated health.

The five chronic-prevalent diseases studied were associated with a poorer HSP. Ischemic heart disease appeared to be the most influential of all of them, with participants being 4 times more likely to present poor HSP if they experienced ischemic heart disease (OR, 4.03; 95% CI 2.37–6.86). The other diseases also showed a statistically significant relationship with poor HSP: diabetes mellitus (OR, 3.77; 95% CI 2.74–5.19), cancer (OR, 3.32; 95% CI 2.02–5.44), depression (OR, 3.31; 95% CI 2.59–4.21), and anxiety (OR 2.14; 95% CI 1.67–2.74).

In the multivariate analysis, age (OR, 1.03; 95% CI 1.02–1.04), higher BMI (OR, 1.05; 95% CI 1.02–1.08) and presenting a prevalent disease [depression (OR, 2.29; 95% CI 1.70–3.09), cancer (OR, 2.08; 95% CI 1.20–3.62), diabetes mellitus (OR, 2.02; 95% CI 1.41–2.90), ischemic heart disease (OR, 1.99; 95% CI 1.09–3.61), and anxiety (OR, 1.68; 95% CI 1.23–2.31)] were associated with a poor HSP. Sex, moderate physical activity, and tobacco consumption showed no significant effect on HSP.

### Relationship between health self-perception and mortality

**FIGURE 1:**
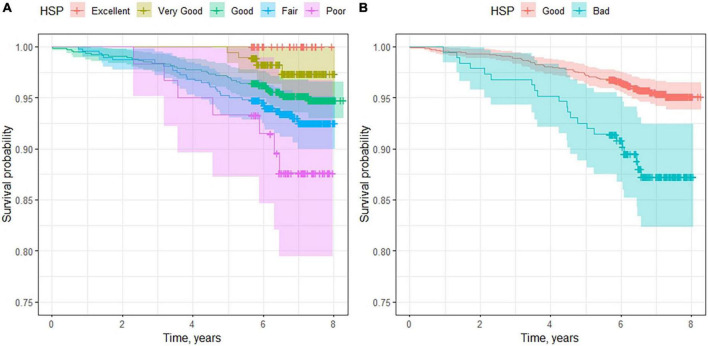
Survival curves for patients according to their self-rated health. In **(A)**, we consider the five groups defined in the SF-36 questionnaire. **(B)** Shows the survival curves for good and poor health self-perception.

Over the course of the follow-up, 78 participants died (5.1%) (Table 3). In the patient group who rated their health as poor, 7.2% died, while in the group who reported a good HSP, only 4% died. Figure 1 shows the survival curves for the five groups of responses to the SF-36 questionnaire and for the grouping by good and poor HSP. As can be observed, all of the participants who described their health as excellent were still alive by the end of the follow-up, while the survival of those who stated having poor health was 88%. For the groups with good and poor HSP, there were also statistically significant differences between their estimated survival curves (*P* log-rank test < 0.01).

**TABLE 3:** Demographics, lifestyle, and comorbidities for patients at 5–8 years of follow-up.

	Alive *n* = 1438	Exitus *n* = 78	*P*
Age, mean (SD)	51.5 (17.1)	73.2 (10.9)	< 0.001
Sex			0.024
Male, n (%)	633 (44.0)	45 (57.7)	
Female, n (%)	805 (56.0)	33 (42.3)	
HSP			0.009
Good, n (%)	937 (65.1)	39 (50.0%)	
Poor, n (%)	501 (34.9)	39 (50.0%)	
BMI, kg/m^2^	28.1 (5.0)	29.9 (5.5)	0.007
Physical activity			0.009
Low, n (%)	557 (38.7)	39 (50.0)	
Mid, n (%)	521 (36.2)	31 (39.7)	
High, n (%)	360 (25.1)	8 (10.3)	
Smoking status			0.008
No, n (%)	784 (54.5)	41 (52.6)	
Ex, n (%)	365 (25.4)	30 (38.5)	
Current, n (%)	289 (20.1)	7 (8.9)	
Alcohol			0.588
0–9 g/week, n (%)	515 (35.8)	31 (39.7)	
10–139 g/week, n (%)	573 (39.9)	25 (32.0)	
140–279 g/week, n (%)	227 (15.8)	14 (18.0)	
>280 g/week, n (%)	123 (8.6)	8 (10.3)	
DM, n (%)	56 (11.5)	22 (28.2)	< 0.001
IHD, n (%)	50 (3.5)	15 (19.3)	< 0.001
Cancer, n (%)	57 (3.9)	14 (17.9)	< 0.001
Depression, n (%)	218 (15.16)	10 (12.8)	0.688
Anxiety, n (%)	319 (22.3)	15 (19.5)	0.663

BMI, body mass index; DM, diabetes mellitus; IHD, ischemic heart disease; HSP, self-rated health.

**FIGURE 2:**
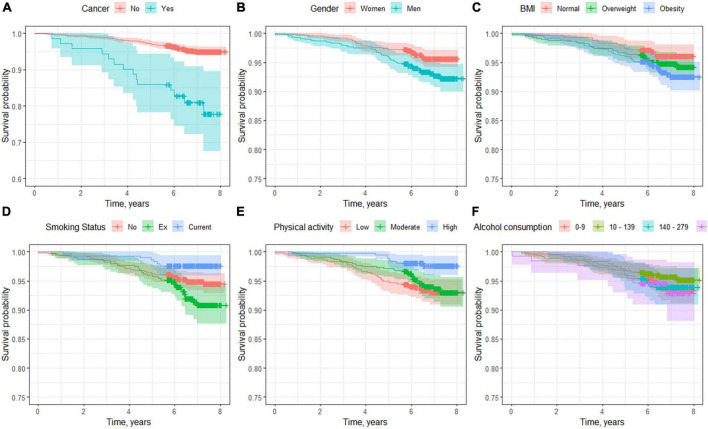
Survival curves according to demographic factors and lifestyles. **(A)** Age, **(B)** sex differences, **(C)** body mass index classification, **(D)** smoking status, **(E)** physical activity, and **(F)** alcohol consumption.

Figure 2 shows the effects of the demographic variables and lifestyle on mortality. In addition to HSP, we can observe a higher percentage of deceased among the male sex (6.6%) than among the female sex (3.9%). The older population had more deaths, with reduced survival in the older ages. The men had a mortality risk 73% greater than that of the women (HR, 1.73; 95% CI 1.10–2.71), and for each year of life, the risk increased by 10% (HR, 1.10; 95% CI 1.08–1.12). For each unit increase in BMI, the mortality risk increased by 6% (HR, 1.06; 95% CI 1.02–1.10). Performing physical activity was a protective factor for mortality, reducing the risk 67% (HR, 0.33; 95% CI 0.15–0.71) for high activity and 24% for moderate activity (HR, 0.86; 95% CI 0.53–1.38). Lifestyle habits such as the consumption of tobacco or alcohol showed no greater risk of mortality in this follow-up period.

**FIGURE 3:**
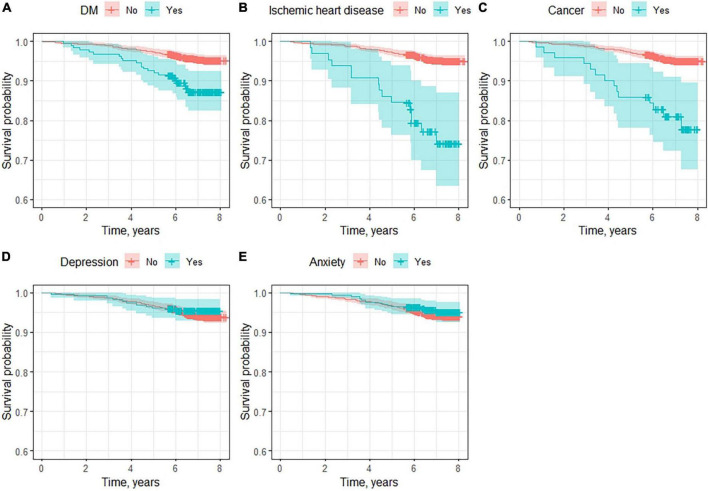
Survival curves for main comorbidities. **(A)** Diabetes mellitus, **(B)** ischemic heart disease, **(C)** cancer, **(D)** depression, and **(E)** anxiety.

With regard to comorbidities (Figure 3 and Table 4), ischemic heart disease (HR, 5.82; 95% CI 3.31–10.20) was the most influential disease, increasing the mortality risk by more than 5 times compared with those who did not experience it. In second place was cancer (HR, 4.6; 95% CI 2.61–8.29), followed by diabetes mellitus (HR, 2.90; 95% CI 1.83–4.59). In terms of mental diseases, depression (HR, 1.34; 95% CI 0.86–2.23) and anxiety (HR, 0.82; 95% CI 0.47–1.45) showed no significant association.

**TABLE 4:** Univariate and adjusted hazard ratios for exitus and their 95% confidence interval.

	Univariate analysis		Adjusted for age		Multivariate model	
			
	HR	95% CI	HR	95% CI	HR	95% CI
Age	1.10*	1.08–1.12	–	–	1.10*	1.08–1.13
Poor HSP	2.08*	1.30–3.32	1.03	0.64–1.65	0.95	0.55–1.61
Male sex	1.57	0.98–2.52	1.98*	1.08–1.23	1.91	1.04–3.51
BMI	1.05*	1.01–1.09	1.01	0.95–1.06	1.00	0.94–1.06
Physical activity						
Low	Ref.		Ref.		Ref.	
Intermediate	0.78	0.47–1.28	0.94	0.56–1.56	0.82	0.48–1.38
High	0.31*	0.14–0.70	0.63	0.29–1.37	0.46	0.21–1.03
Smoking						
No	Ref.		Ref.		Ref.	
Ex	1.52	0.93–2.49	1.99*	1.20–3.31	1.26	0.65–2.44
Current	0.34*	0.15–0.78	2.63*	1.14–6.07	1.80	0.73–4.48
Alcohol						
0–9 g/week	Ref.		Ref.		Ref.	
10–139 g/week	0.92	0.53–1.60	1.11	0.64–1.91	1.03	0.59–1.81
140–279	1.07	0.55–2.09	0.74	0.37–1.48	0.53	0.25–1.11
g/week	0.98	0.44–2.18	0.97	0.41–2.26	0.70	0.27–1.81
> 280 g/week						
DM	2.85*	1.76–4.63	1.26	0.76–2.09	1.13	0.66–1.96
IHD	6.83*	3.81–12.26	2.41*	1.31–4.43	1.73	0.80–3.73
Cancer	5.18*	2.85–9.41	1.97*	1.05–3.72	1.56	0.78–3.11
Depression	0.87	0.43–1.76	0.62	0.30–1.28	0.74	0.34–1.57
Anxiety	1.02	0.56–1.84	1.12	0.63–2.00	1.30	0.71–2.39

*Indicates statistically significant odds ratios for *p* < 0.05. BMI, body mass index; DM, diabetes mellitus; HR, hazard ratio; IHD, ischemic heart disease; HSP, self-rated health.

Once the age-adjusted analysis was performed (Table 4), several of the variables lost their significant association with mortality: HSP (HR, 0.95; 95% CI 0.61–1.49), BMI (HR, 1.02; 95% CI 0.97–1.07), a high level of physical activity (HR, 0.59; 95% CI 0.28–1.27) and developing diabetes mellitus (HR, 1.40; 95% CI 0.88–2.23). The individuals who smoked (HR, 2.61; 95% CI 1.10–6.18) and the ex-smokers (HR, 2.01; 95% CI 1.25–3.22) were associated with greater mortality. The male sex (HR, 1.99; 95% CI 1.27–3.12), ischemic heart disease (HR, 2.29; 95% CI 1.29–4.07), and cancer (HR, 1.94; 95% CI 1.08–3.48) maintained statistical significance and were associated with a greater mortality risk.

Lastly, after adjusting for all the potential confounders, the only variable that maintained its significance level in this study was age (HR, 1.10; 95% CI 1.07–1.12). The rest of the variables, which included HSP (HR, 0.82; 95% CI 0.50–1.33), were not associated with survival.

## Discussion

This follow-up study of more than 5 years conducted on a representative sample of the adult population found that a poorer HSP is more likely in older individuals, those with a higher BMI and in carriers of a chronic or mental disease. All of these have been recognized as determinants of HSP through a multivariate analysis. Moreover, both moderate alcohol consumption (10–139 g/week) and the weekly performance of a high level of physical activity have been related to a better HSP among the participants. We also found a statistically significant association between HSP and the patients’ emotional state (anxiety and depression) after adjusting for other potentially confounding clinical variables. Finally, we report an association between HSP and mortality, with higher mortality rates in those individuals who indicated poorer HSP. However, after adjusting for the potential confounders, such as chronic morbidity, lifestyles, and especially age, there was no statistically significant association between HSP and mortality.

With regard to the relationship between HSP and mortality, other studies had seen association also after adjusting for potential confounders (e.g., 13, 10, 21, and 22). In fact, a number of studies have shown that self-rated health is an independent predictor and, in terms of early mortality, is in second place as a predictor, behind age (21). The results of studies are variable, but there is also differences on sampling strategies, study population, HSP assessment, follow-up period, and statistical analysis. In our case, the results suggest that a poor HSP is indicative of mortality in the general population, but this effect is mediated mainly by age. These discrepancies in the results are likely due mainly to that fact that, unlike our study, most of the studies had participants older than 60–65 years. In a study with a broad representative sample of the population between the ages of 25 and 74 years, Idler et al. (12) also found no association between HSP and mortality. The effect of HSP on survival appears to occur only in the older adult group. Thus, in the meta-analysis performed by DeSalvo et al. (5), the studies that showed an increase in mortality risk in the individuals with poor HSP were those in which their participating were older than 60 years.

The effect that HSP exerts on mortality appears to decrease over the course of time. A recent study by Lorem et al. (23) reported a reduction in the HR of HSP, adjusted for confounders, for follow-up times longer than 5 years.

Other studies on the determinants of a poor HSP have found similar results. Tamayo-Fonseca et al. (13) analyzed both sexes separately, observing an association between age, restricted mobility, educational level, the previous use of hospitals, and the presence of chronic diseases. As with our study, the authors found that the variable that most affected HSP is having a chronic disease. Ge et al. (24) specifically analyzed the individual influence of certain chronic diseases on HSP. The negative impact of comorbidities could be related to the fact that these diseases usually restrict mobility and the performance of daily activities and increase the prevalence of chronic pain. However, neither diabetes mellitus nor ischemic heart disease showed a statistically significant effect in their study. With regard to mental illness, our results agree with those in the literature (25–27) in that a current or past history of depressive symptoms negatively affects HSP.

One of the triggers of these diseases in our society is low financial income (28); however, our study was unable to take this factor into account when analyzing the results. The literature shows how a higher income rate could reduce stress levels and in turn improve the individual’s overall satisfaction. Within the cardiovascular diseases, a higher income could also have greater influence, because individuals with greater purchasing power could invest more in healthy eating, sports activities and leisure time (3).

In terms of study limitations, we should first note that, in the initial part of the study (the association between HSP and demographic variables), we could not establish a clear line of causality given that we used a cross-sectional approach. Another possible limitation of the study was not incorporating other variables that might have affected the research, such as social (financial level, unemployment, educational level, support level, and psychosocial problems) and physical factors (limited mobility and other diseases). The follow-up was limited to 5–8 years, with a mortality rate of 5.1%. Given the small sample size, the association between mortality and HSP could have been compromised. Lastly, we should note that the voluntary participation in the study could have caused a bias. Although this a population-based study and individuals were sampled from the community, they could consent or refuse to participate in research and their willingness to participate is unlikely to be random. Individuals with more severe conditions or a poorer HSP might have declined to participate in the study. Both the mortality data and the poor HSP might have been underestimated because the individuals who had more advanced stages of disease were not included in the study. Finally, this study was performed in a single municipality, so the generalization of the results could be compromised. However, in a Estrada municipality we can see a heterogeneous population part rural part urban, with social indicators similar to the whole community. Thus, we consider this municipality as representative of Galician people.

## Conclusion

1) Health self-perception is associated with age, lifestyle, and certain prevalent diseases.
2) A poorer HSP is associated with increased mortality, but this predictive capacity disappeared after adjusting for potential confounders such as age, lifestyle, and prevalent diseases.
3) Health self-perception is a global measure of health status that must be taken into account when evaluating patients. It is associated with prevalent diseases as diabetes mellitus, ischemic heart disease, and anxiety after adjusting for possible cofounders.

## Data availability statement

The raw data supporting the conclusions of this article will be made available by the authors, without undue reservation.

## Ethics statement

The studies involving human participants were reviewed and approved by Galician Clinical Research Ethics Committee, Santiago de Compostela, Spain (CEIC, reference number: 2012-025). The patients/participants provided their written informed consent to participate in this study.

## Author contributions

CF-M wrote the manuscript and collaborated in the study design. AC-C conducted the patient interviews and data collection. JS-C conducted the patient interviews and their follow-up. OR-S wrote Introduction and Discussion section. ÓL-B conducted statistical analysis concerning survival models. MF-D conducted the patient interviews. FG conducted statistical analysis of variables influencing self-perceived health and provided the curated data for the study. All authors contributed to the article and approved the submitted version.
